# Pediatric Kidney Transplantation: Frameshift in Medical and Surgical Management. Does the Perioperative Setting Have an Impact on Transplant Outcome? A Single-Center Experience

**DOI:** 10.3389/fsurg.2022.881494

**Published:** 2022-05-02

**Authors:** Berenice Bergel, Tamara Geppert, Beatriz Bañuelos Marco, Frank Friedersdorff, Dominik Müller, Caroline Kempf, Nils Lachmann, Anja Lingnau

**Affiliations:** ^1^Department of Urology, Charité – Universitätsmedizin Berlin, Berlin, Germany; ^2^Evangelisches Krankenhaus Königin Elisabeth Herzberge, Berlin, Germany; ^3^Department of Pediatric Gastroenterology, Berlin Institute of Health, Nephrology and Metabolic Disorders, Charité – Universitätsmedizin Berlin, Humboldt-Universität zu Berlin, Berlin, Germany; ^4^Berlin Institute of Health, Institute of Transfusion Medicine, Charité – Universitätsmedizin Berlin, Humboldt-Universität zu Berlin, Berlin, Germany

**Keywords:** pediatric renal transplantation, kidney, living donor, PRA, panel reaktive antibodies, CAKUT

## Abstract

**Introduction:**

Frameshift in medical management as well as in surgical thinking is putting the patient as a whole is the focus, rather than just the disease. To optimize the treatment of our pediatric transplant patients in our institution, we changed in 2013 the transplant program setting, treating, and operating all patients with pediatric transplant exclusively in a pediatric environment. The aim of this study was to analyze whether or not this change had an impact on patients safety, patient population, and patients and transplant outcome.

**Methods:**

In the retrospective analysis, we compared transplant outcome of two eras. Era1 (2008–2012) solely included patients treated in the adult facilities, era 2 (2013–2017) patients were exclusively treated in the pediatric environment.

**Results:**

There were 53 patients with renal transplant, with era 1 (28 patients) and era 2 (25 patients). Overall mortality was 5.6%. Median recipient age at transplantation was 13.2 years in era 1 and 8.59 years in era 2, median recipient weight at transplantation was 41.7 kg in era 1 vs. 26 kg in era 2, median size 149. 5 cm (era 1) vs. 123 cm in era2 (*p* = 0.05). The direct recipient/donor weight ratio remained stable in both eras, for recipients below 20 kg we saw a larger weight mismatch in era 1 (0.84 vs. 0.66). In the subgroup of patients with congenital anomalies of the kidney and urinary tract (CAKUT) those were significantly younger at onset of dialysis (*p* < 0.001) and at time of transplantation (*p* < 0.001), also they were less in body weight (*p* < 0.01), and body size (*p* < 0.001), this subgroup was larger in era 2. HLA mismatch data, serum creatinine, and GFR yield comparable results in both groups. Median time to detection of DSA was 46.2 month (3.8 years).

**Conclusion:**

Since children with ESRD at the time of transplant trend to be younger and smaller, it is crucial to ensure a medical environment that is able to address their particular challenges. Even in this recipient cohort, renal transplantation can be performed safely as outlined by our data.

## Introduction

Renal transplantation remains the treatment of choice for children with end stage renal disease (ESRD). It confers improved survival (1), growth (2), and health-related quality of life (3) compared to dialysis. Due to new immunosuppressive medication, decreased number of rejections and improvement of transplant- and patient outcome is seen.

However, since the first pediatric renal transplant in the 1960‘s the field of pediatric renal transplantation is still evolving in many fields like immunosuppression, surgical techniques, pre-, peri-, and postoperative care, donor-recipient matching and patients and donor selection, and medical management with a frameshift also in surgical thinking putting the patient as a whole is the focus, rather than just the disease (4, 5).

To optimize the treatment of the pediatric patient population in our institution, we changed in 2013 the transplant program setting.

Pediatric renal transplants were performed over a long period in the general urology and high-volume transplant setting. Pediatric transplant patients were transported to the site of transplantation, postoperative care was performed in adult intensive care and when patients were stable enough, they were transported back to the pediatric facilities, which were at a different hospital site across town.

In 2013, the decision was made to change this setting to save the children, the transportation and pediatric transplantations were since then solely carried out in the pediatric hospital setting. Pre- and postoperative care was performed in pediatric nephrology, pediatric anesthesia, pediatric intensive care, and surgery in pediatric operating theater mostly by pediatric urologists. Also pre-transplantation patient evaluation was performed by the pediatric team (urology, nephrology, and anesthesia).

The aim of this study was to analyze whether or not this change had an impact on patients safety, patient population, and patients and transplant outcome.

## Patients and Methods

This study is a retrospective analysis. We reviewed medical records of all patients and collected data on medical history, clinical, and radiological findings in a database with standardized variables. Our database included pediatric renal transplantations (0–18 years) from January 2008 to December 2017. We decided to select patients from this era (the last 5 years in the adult setting and the first 5 years in the pediatric setting) mainly due to the fact that in this period of time the immunosuppression maintenance included steroids, mycophenolatmofetil (MMF), and tacrolimus as standard in all patients.

In Era1 (2008–2012) solely included patients treated in the adult facilities, in era2 (2013–2017) patients were exclusively treated in the pediatric environment.

We evaluated preoperative parameters: patients and donors demographic data (age, body weight and height, patient's primary disease, type, and duration of dialysis, HLA-A, B and DR mismatch (according to the EUROTRANSPLANT matching criteria), intraoperative parameters such as operating technique, intraoperative complications, ischemia time, and postoperative parameters as function of transplant, serum creatinine and glomerular filtration rate (GFR), calculated according to the Schwartz formula (6), and duration of hospitalization, panel reactive antibodies (PRA) (solid phase assay, pre-transplantation, and in follow-up assessments post-transplantation, donor specific antibodies (DSA) (LABScreen® Mixed and Single Antigen Beads (OneLambda, West Hills, CA, USA)(7). Samples were measured on a Luminex® 200 (Luminex®, Austin, TX, USA) and analyzed using the HLA Fusion software (OneLambda).

Information from the hospital archive, electronic medical records, and EUROTRANSPLANT were used for extracting the data.

## Statistical Analyses

SPSS 22 (IBM Corporation, Somers, NY, USA) was used to perform statistical analyses. Non-parametric tests (Mann–Whitney U test, Kruskal–Wallis test) were performed. The chi-square test was used to evaluate categorical variables. A *p*-value of < 0.05 was considered significant.

## Results

There were 53 patients with renal transplant altogether, in era1 (2008–2012) were 28 patients and era2 (2013–2017) 25 patients, respectively.

Overall mortality was 1, 9%, 0% in era1, and 4% (*n* = 1) in era 2 (patient died of multi organ failure due to sepsis 1 month after transplantation).

The number living donor transplantations was higher in era 2 (44 vs. 32.1% in era 1), also in era 2 were more boys (64 vs. 46.5% in era 1).

Mean follow up time for transplant outcome was minimally 3 years in both groups, for era 1 longer follow up data is available, this applied for donor specific antibody (DSA) measurements.

**Figure 1:**
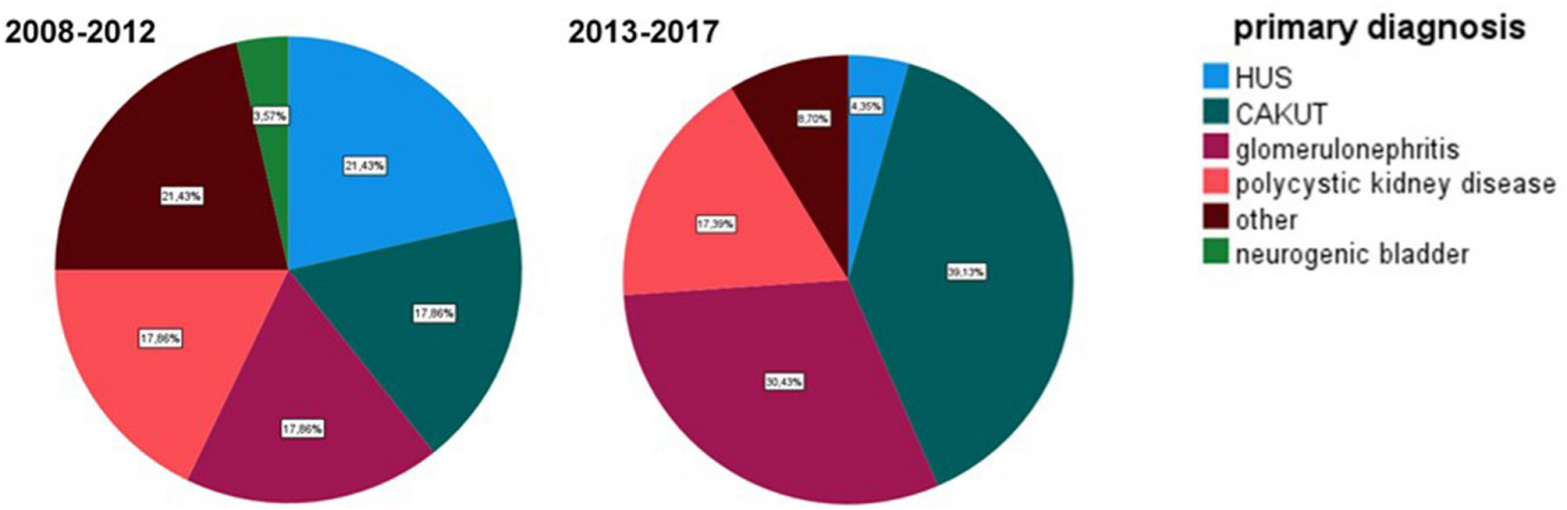
Primary diagnosis in the analyzed eras. Era 1 2008–2012, era 2 2013–2017.

Leading causes for ESRD was in era 1 the hemolytic uremic syndrome (HUS) (21.4%), unspecified reasons such as stones, vasculitis, and postnatal hypoxia (21.4%) followed by congenital disorders of the kidneys and the urinary tract (CAKUT) (17.8%). Primary diagnosis in era 2 was CAKUT (39.1%, glomerulonephritis (30.4%) and polycystic kidney disease (ADPKD) (17.3%) Figure 1.

Demographic data are outlined in Table 1.

**Table 1:** Demographic data of donor and recipients, HLA mismatches and ischemia time.

		**Median**	**Mean ±SD**	***p-*value**
**Recipient (parameters at time of transplant)**				
**Age in years**				
	2008–2012	13,27	11,62 ± 5	
	2013–2017	8,59	9,35 ± 5	0.12
**Body weight in kg**				
	2008–2012	41.7	38.23 ± 16.19	
	2013–2017	26	27.88 ± 13.94	0.175
**Body size in cm**				
	2008–2012	149.5	139.53 ± 27,81	
	2013–2017	123	124.8 ± 27.93	0.05
**Age at onset of dialysis**				
	2008–2012	11.54	9.68 ± 5.2	
	2013–2017	6.89	7.83 ± 5.5	0.31
**Days on dialysis**				
	2008–2012	526	678 ± 527	
	2013–2017	651	621 ± 606	0.47
**Waiting time for organ in months**				
	2008–2012	6.2	16.11 ± 21.12	
	2013–2017	5.1	11.25 ± 16.98	0.22
**Hospital stay after operation in days**
	2008–2012	24	24.71 ± 7,2	
	2013–2017	23.3	24.88 ± 6,7	0.78
**Donor**				
**Donor age in years**				
	2008–2012	40	29.9 ± 17.9	
	2013–2017	24	22.7 ± 16.5	0.11
**Donor weight in kg**				
	2008–2012	62	61.5 ± 26.3	
	2013–2017	65	56.2 ± 26.3	0.47
**Ratio weight recipient/donor**				
	2008–2012	0.57	0,67 ± 0.28	
	2013–2017	0.54	0,68 ± 0.57	0.21
**HLA mismatch**				
HLA-A,B,DR	2008–2012	3	3.14 ± 1.5	
	2013–2017	3	3.3 ± 1.34	0.77
HLA-DR	2008–2012	1	1.1 ± 0.68	
	2013–2017	1	1.08 ± 0.49	0.8
**Cold ischemia time in min**				
	2008–2012	626	582 ± 72.55	
	2013–2017	505	505 ± 63.37	0.41
**HLA antibodies positive in %**				
Pre-transplant	2008–2012	17,85		
	2013–2017	28		0.38
Post-transplant	2008–2012	71.42	
	2013–2017	40		n/a (longer f up)
**DSA positive in %**				
Pre-transplant	2008–2012	3.57		
	2013–2017	0		0,345
Post-transplant	2008–2012	42.85		
	2013–2017	8		n/a (longer fu)

Median recipient age at transplantation was 13.2 years in era1 and 8.59 years in era2, median recipient weight at transplantation was 41.7 kg in era1 vs. 26 kg in era2, median size 149.5 cm (era 1) vs. 123 cm in era 2 (*p* = 0.05). The direct recipient/donor weight ratio remained stable in both eras, for recipients below 20 kg we saw a larger weight mismatch in era1 (0.84 vs. 0.66).

Patients in era 2 were also younger when dialysis was initiated (median 11.5 years era 1 vs. 6.89 years in era 2) and spent more time on dialysis (526 days in era 1 and 651 days in era 2). The number of HLA mismatches and cold ischemia time showed no significant difference amongst the eras (data is also shown in Table 1).

Data for PRA pre- and post-transplantation and DSA pre- and post-transplantation is shown in Table 1. Since DSA post-transplantation were more frequent in era 1. We used the longer follow up data for this group and found that median time to detection of DSA was in this cohort 46.2 month (3.8 years).

Serum creatinine at time of dismissal, 1 and 3 years after transplantation was for era 1 0.89 ± 0.45 mg/dl, 1.35 ± 1.9 mg/dl and 1.5 ± 1.8 mg/dl and for era 2 0.67 ± 0.27 mg/dl, 0.91 ± 0.65 mg/dl, and 0.87 ± 0.37 mg/dl, respectively.

GFR at time of dismissal, 1 and 3 years after transplantation was for era 1 75.7 ± 25.25 ml/min, 67.15 ± 20.68 ml/min and 61.57 ± 24.30 ml/min and for era 2 85.93 ± 27.68 ml/min, 74.20 ± 24.60 ml/min, and 76.36 ± 21.58 ml/min, respectively.

**Figure 2:**
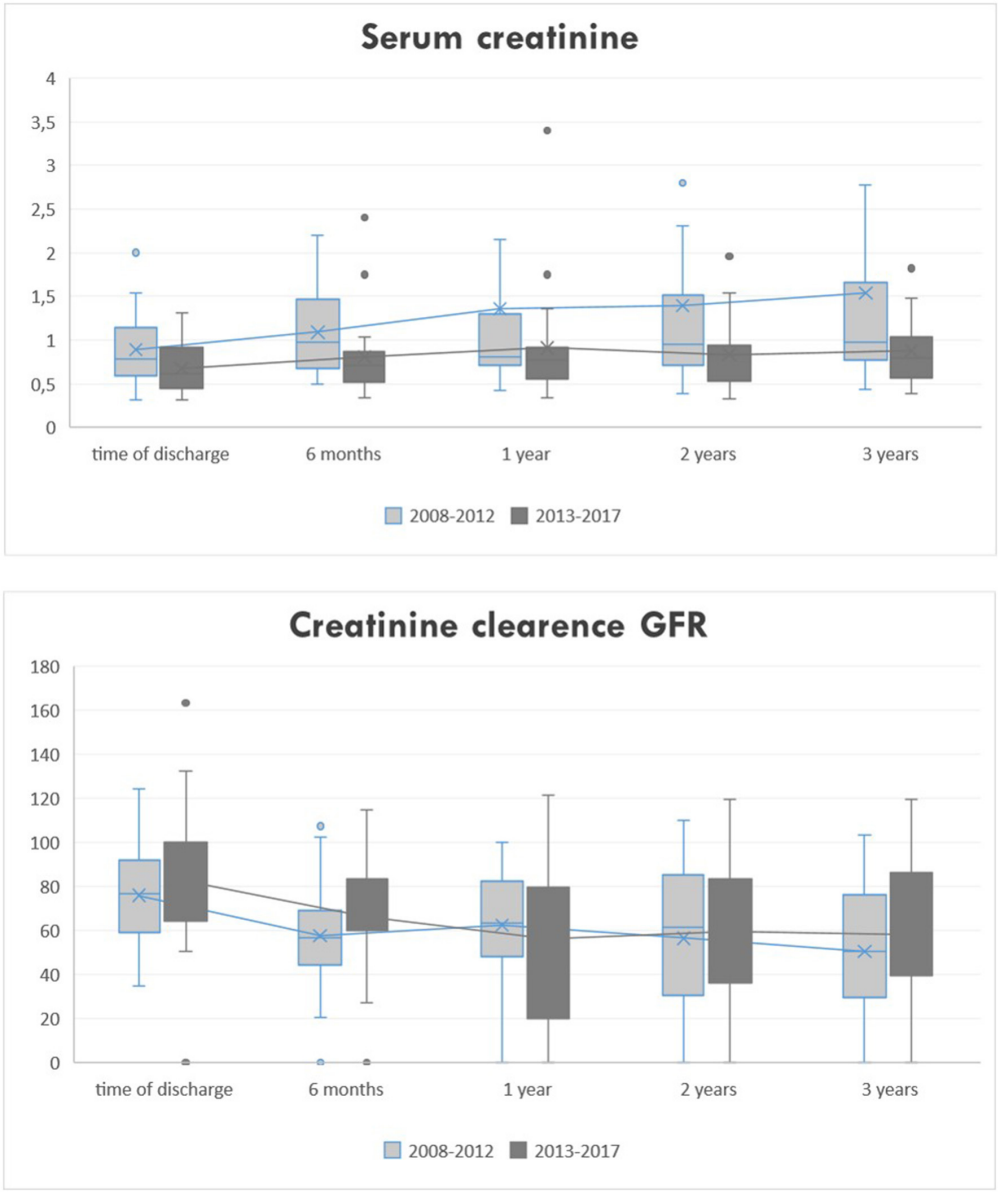
Clinical course of serum creatinine and GFR.

The full courses for serum creatinine and GFR over 3 years are shown in Figure 2.

**Figure 3:**
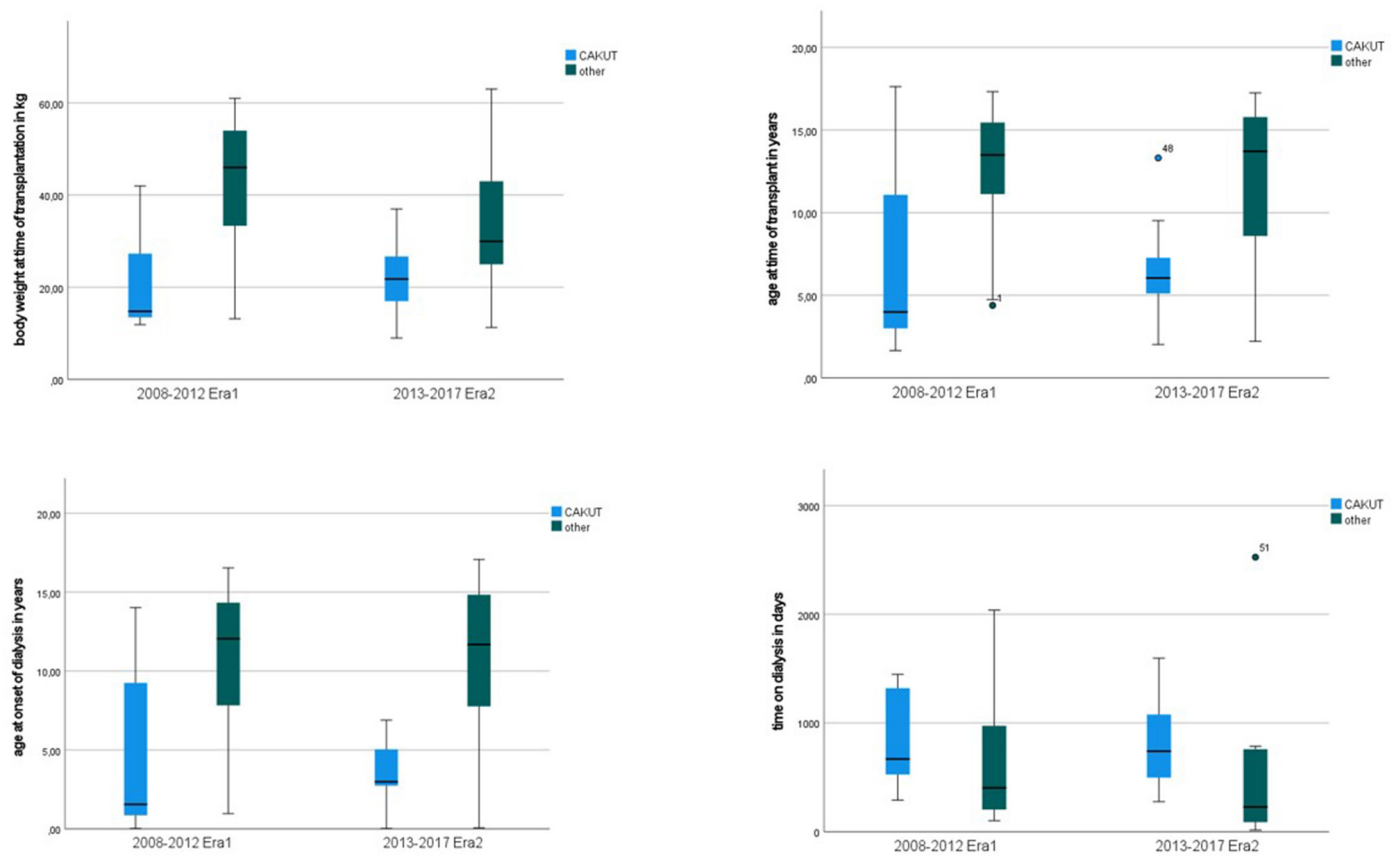
Recipients age at onset of dialysis and at time of transplant according to primary diagnosis.

Since demographic data revealed differences in recipient's characteristics (primary disease, age, size and body weight at time of transplantation) we further analyzed this data and found that the subgroup of patients with CAKUT as primary diagnosis were significantly younger at onset of dialysis (*p* < 0.001) and at time of transplantation (*p* < 0.001), also they were less in body weight (*p* < 0.001), and body size (*p* < 0.001); see Figure 3.

## Discussion

Outcome after pediatric kidney transplantation continuously improved over the last decades (8). Also, children are assigned additional waiting points in the Eurotransplant Kidney Allocation System (ETKAS) to equalize imbalances in organ exchange (9). Many transplant programs worldwide have a similar approach and prioritize children in solid organ allocation since children seem to benefit from this organ for a longer period of time (10). Therefore, constant evaluation of transplant outcome data remains crucial. In the present study, we wanted to know, whether pediatric renal transplantation outside of an adult transplantation unit with high volume throughput is a safe option for pediatric renal transplantation. Our Data clearly shows that this is the case. Results in patient, graft survival and graft function were excellent, improved over the years and are in line with the literature (8).

Our data also shows that the pediatric patient pool did not remain stable over the years. One aspect is the trend to transplant children at a younger age, which can also be seen in the NAPRTCS (North American Pediatric Renal Trials and Collaborative Studies) data (8). For small recipients (below 20 kg) we were accepting smaller donors in era 2 with equally good results. Recently published data support this approach in utilizing small donor kidneys for small recipients (11). In our study, we found a very big subgroup of patients with HUS as primary diagnosis in era 1 (2008–2013). In 2011, an *Escherichia coli* strain caused in Germany an outbreak of >800 cases of HUS, including 90 children. However, since most of the children recovered in the intermediate follow up, they were not found in the patient pool in our cohorts (12). We can only speculate that advances in treatment for pediatric HUS with better renal outcome reduced the number of these patients in the second era (13).

In developed countries, CAKUT and is the main cause for ESRD in children (14). Our data shows that children with CAKUT as primary diagnosis are significantly younger and smaller at onset of dialysis and at time of transplantation, which is a discussed topic in the literature (15). One might speculate that the shift of primary diagnosis might be a direct result of improved survival of these children in the postnatal period. Further epidemiological data needs to be collected.

Pediatric interdisciplinary care at any time during in-patient stay at renal transplantation in era 2 leads to comparable outcome to era 1 though recipients were even younger and thus had a lower body weight. Encouraging data of other studies and of our cohort in era 2 might support efforts to offer smaller donor kidneys aiming for shorter waiting time but with comparable outcome of patients with pediatric renal transplant in the future.

## Conclusion

Since children with ESRD at the time of transplant trend to be younger and smaller over the years; it is crucial to ensure a medical environment that is able to address their particular challenges in perioperative care according to their individual needs. Even in this more challenging recipient cohort, renal transplantation can be performed safely as outlined by our data.

## Data Availability Statement

The raw data supporting the conclusions of this article will be made available by the authors, without undue reservation.

## Ethics Statement

The studies involving human participants were reviewed and approved by Charité Universitätsmedizin Berlin Ethikkomission Campus Virchow Klinikum Antrag EA2/026/22. Written informed consent to participate in this study was provided by the participants' legal guardian/next of kin.

## Author Contributions

BBe, AL, TG, and CK had substantial contributions in data acquisition and design of the work. BBa, FF, DM, and AL were responsible for conception and interpretation of the data for the work. All authors revised the work critically for intellectual content and gave final approval for publication and are accountable for all aspects of the work.

## Conflict of Interest

NL reports personal fees from BmT GmbH, outside the submitted work. The authors declare that the research was conducted in the absence of any commercial or financial relationships that could be construed as a potential conflict of interest.

## Publisher's Note

All claims expressed in this article are solely those of the authors and do not necessarily represent those of their affiliated organizations, or those of the publisher, the editors and the reviewers. Any product that may be evaluated in this article, or claim that may be made by its manufacturer, is not guaranteed or endorsed by the publisher.

## Abbreviations

ESRD: end-stage renal disease
LD: living donor
DD: deceased donor
GFR: glomerular filtration rate
HUS: hemolytic uraemic syndrome
PRA: panel reaktive antibodies
DSA: donor specific antibodies
CAKUT: congenital anomalies of the kidney and urinary tract.
